# Supplementary material for: Food Beliefs and the Risk of Orthorexia in Patients with Inflammatory Bowel Disease
Source: Nutrients. 2024 Apr 17;16(8):1193. doi: 10.3390/nu16081193 (PMC11054879; doi:10.3390/nu16081193)
Supplement: Supplementary file 1 [file nutrients-16-01193-s001.zip › nutrients-2926594-supplementary.pdf]

Supplementary table n.1

	Patients with risk of orthorexia N=87			
**Questions**	**Always**	**Often**	**Sometimes**	**Never**
**1**	5 (5.7%)	10 (11.5%)	14 (16.1%)	58 (66.7%)
**2**	0	5 (5.7%)	6 (6.9%)	76 (87.4%)
**3**	7 (8%)	12 (13.8%)	17 (19.5%)	51 (58.6%)
**4**	14 (16.1%)	23 (26.4%)	36 (41.4%)	14 (16.1%)
**5**	17 (19.5%)	3 (3.4%)	24 (27.6%)	43 (49.4%)
**6**	50 (57.5%)	9 (10.3%)	23 (26.4%)	5 (5.7%)
**7**	2 (2.3%)	16 (18.4%)	7 (8%)	62 (71.3%)
**8**	6 (6.9%)	34 (39%)	36 (41.4%)	11 (12.6%)
**9**	11 (12.6%)	17 (19.5%)	24 (27.6%)	35 (40.2%)
**10**	22 (25.3%)	16 (18.4%)	30 (34.4%)	19 (21.8%)
**11**	20 (23%)	22 (25.3%)	18 (20.7%)	27 (31%)
**12**	36 (41.4%)	22 (25.3%)	18 (20.7%)	11 (12.6%)
**13**	9 (10.3%)	6 (6.9%)	13 (14.9%)	59 (67.8%)
**14**	45 (51.7%)	33 (37.9%)	5 (5.7%)	4 (4.6%)
**15**	0	5 (5.7%)	16 (18.4%)	66 (75.9%)

Supplementary table n.2

	Patients without risk of orthorexia N=26			
**Questions**	**Always**	**Often**	**Sometimes**	**Never**
**1**	1 (3.8%)	1 (3.8%)	5 (19.2%)	19 (73.1%)
**2**	0	0	1 (3.8%)	25 (96.2%)
**3**	0	0	6 (23%)	20 (77%)
**4**	2 (7.7%)	1 (3.8%)	14 (53.8%)	9 (34.6%)
**5**	9 (34.6%)	5 (19.2%)	3 (11.5%)	9 (34.6%)
**6**	6 (23%)	3 (11.5%)	7 (26.9%)	10 (38.5%)
**7**	0	1 (3.8%)	3 (11.5%)	22 (84.6%)
**8**	4 (15.4%)	12 (46.2%)	8 (30.8%)	2 (7.6%)
**9**	2 (7.7%)	5 (19.2%)	5 (19.2%)	14 (53.8%)
**10**	3 (11.5%)	1 (3.8%)	4 (15.4%)	18 (69.2%)
**11**	2 (7.7%)	4 (15.4%)	11 (42.3%)	9 (34.6%)
**12**	0	8 (30.8%)	4 (15.4%)	14 (53.8%)
**13**	2 (7.7%)	3 (11.5%)	9 (34.6%)	12 (46.2%)
**14**	8 (30.8%)	8 (30.8%)	6 (23%)	4 (15.4%)
**15**	1 (3.8%)	0	1 (3.8%)	24 (92.3%)

Supplementary table n.3

	Control group with risk of orthorexia N=21			
**Questions**	**Always**	**Often**	**Sometimes**	**Never**
**1**	4 (19%)	5 (23.8%)	7 (33.3%)	5 (23.8%)
**2**	0	1 (4.8%)	3 (14.2%)	17 (81%)
**3**	0	5 (23.8%)	6 (28.6%)	10 (47.6%)
**4**	1 (4.8%)	7 (33.3%)	9 (42.9%)	4 (19%)
**5**	1 (4.8%)	2 (9.5%)	8 (38%)	10 (47.6%)
**6**	9 (42.9%)	10 (47.6%)	2 (9.5%)	0
**7**	1 (4.8%)	0	3 (14.2%)	17 (81%)
**8**	1 (4.8%)	7 (33.3%)	12 (57%)	1 (4.8%)
**9**	4 (19%)	3 (14.2%)	8 (38%)	6 (28.6%)
**10**	3 (14.2%)	4 (19%)	6 (28.6%)	8 (38%)
**11**	4 (19%)	6 (28.6%)	6 (28.6%)	5 (23.8%)
**12**	8 (38%)	8 (38%)	3 (14.2%)	2 (9.5%)
**13**	2 (9.5%)	3 (14.2%)	11 (52.4%)	5 (23.8%)
**14**	10 (47.6%)	11 (52.4%)	0	0
**15**	0	6 (28.6%)	12 (57%)	3 (14.2%)

Supplementary table n.4

	Control group without risk of orthorexia N=24			
**Questions**	**Always**	**Often**	**Sometimes**	**Never**
**1**	2 (8.3%)	3 (12.5%)	10 (41.7%)	9 (37.5%)
**2**	0	0	2 (8.3%)	22 (91.7%)
**3**	0	0	4 (16.7%)	20 (83.3%)
**4**	0	1 (4.17%)	5 (20.8%)	18 (75%)
**5**	8 (33.3%)	6 (25%)	8 (33.3%)	2 (8.3%)
**6**	5 (20.8%)	7 (29.2%)	9 (37.5%)	3 (12.5%)
**7**	0	0	1 (4.17%)	23 (95.8%)
**8**	8 (33.3%)	10 (41.7%)	6 (25%)	0
**9**	7 (29.2%)	5 (20.8%)	7 (29.2%)	5 (20.8%)
**10**	0	3 (12.5%)	7 (29.2%)	14 (58.3%)
**11**	1 (4.17%)	1 (4.17%)	5 (20.8%)	17 (70.8%)
**12**	5 (20.8%)	7 (29.2%)	6 (25%)	6 (25%)
**13**	2 (8.3%)	2 (8.3%)	9 (37.5%)	11 (45.8%)
**14**	9 (37.5%)	11 (45.8%)	4 (16.7%)	0
**15**	0	2 (8.3%)	15 (62.5%)	7 (29.2%)
